# Supplementary material for: Seeing Inside a Plasmonic Nanogap: Few-Molecule Orientation and Preferential Adsorption
Source: ACS Nano. 2026 Oct 15;20(42):28576–85. doi: 10.1021/acsnano.6c12575 (PMC13718606; doi:10.1021/acsnano.6c12575)
Supplement: Supplementary file 1 [file nn6c12575_si_001.pdf]

Supplementary Information

Seeing inside a Plasmonic Nanogap: Few-molecule Orientation and Preferential Adsorption

Francesco Ciccarello,[1] Nobuaki Oyamada,[1] Thwahira Shirin Alampara,[1] Konstantin Malchow,[1] and Christophe Galland[1,2,*]

[1] *Institute of Physics, Ecole Polytechnique Fédérale de Lausanne (EPFL), CH-1015 Lausanne, Switzerland*

[2] *Center for Quantum Science and Engineering, Ecole Polytechnique Fédérale de Lausanne (EPFL), CH-1015 Lausanne, Switzerland*

Sec. S1. WHY WE NEED SFG TO DISTINGUISH PARALLEL FROM ANTI-PARALLEL MOLECULES

In this section, we discuss what the minimal requirement is to obtain a signal that can distinguish parallel vs. antiparallel arrangements of two sub-systems. An incoherent Raman intensity measurement cannot work, as the projection of the local electric field on a dipole only flips sign upon inversion of the molecule, so the squared modulus is constant. Alternatively, we can look into coherent anti-Stokes Raman scattering (CARS), which is phase-sensitive. This process is described by a third-order nonlinearity, $\chi^{(3)}$. The corresponding third-order nonlinear polarisation, like the linear polarisation, is invariant under the inversion of the system. Indeed, more generally, the n-th order nonlinear transforms as $\chi^{(n)\prime} = (-1)^{n+1}\chi^{(n)}$ under the inversion transformation, represented by the matrix $-I_{3\times3}$. Therefore, even if a phase reference is available, there is no relative phase shift in the signal from a molecule when inversion is applied to it, making it impossible to distinguish the two configurations studied in the main text.

A nonlinear process of even order is required, like second-harmonic or sum-frequency generation, both resulting from a $\chi^{(2)}$ susceptibility. In this case, the second-order polarization flips sign when inversion is applied to the molecule, corresponding to a π phase shift. If the signal interferes with a reference (e.g., another non-inverted molecule or surface, or a local oscillator), then a clear distinction is possible between the two configurations, which is what we prove experimentally in the main text.

* chris.galland@epfl.ch

Sec. S2. NANOSLITS AND NPOS CHARACTERIZATION BY SEM AND FTIR

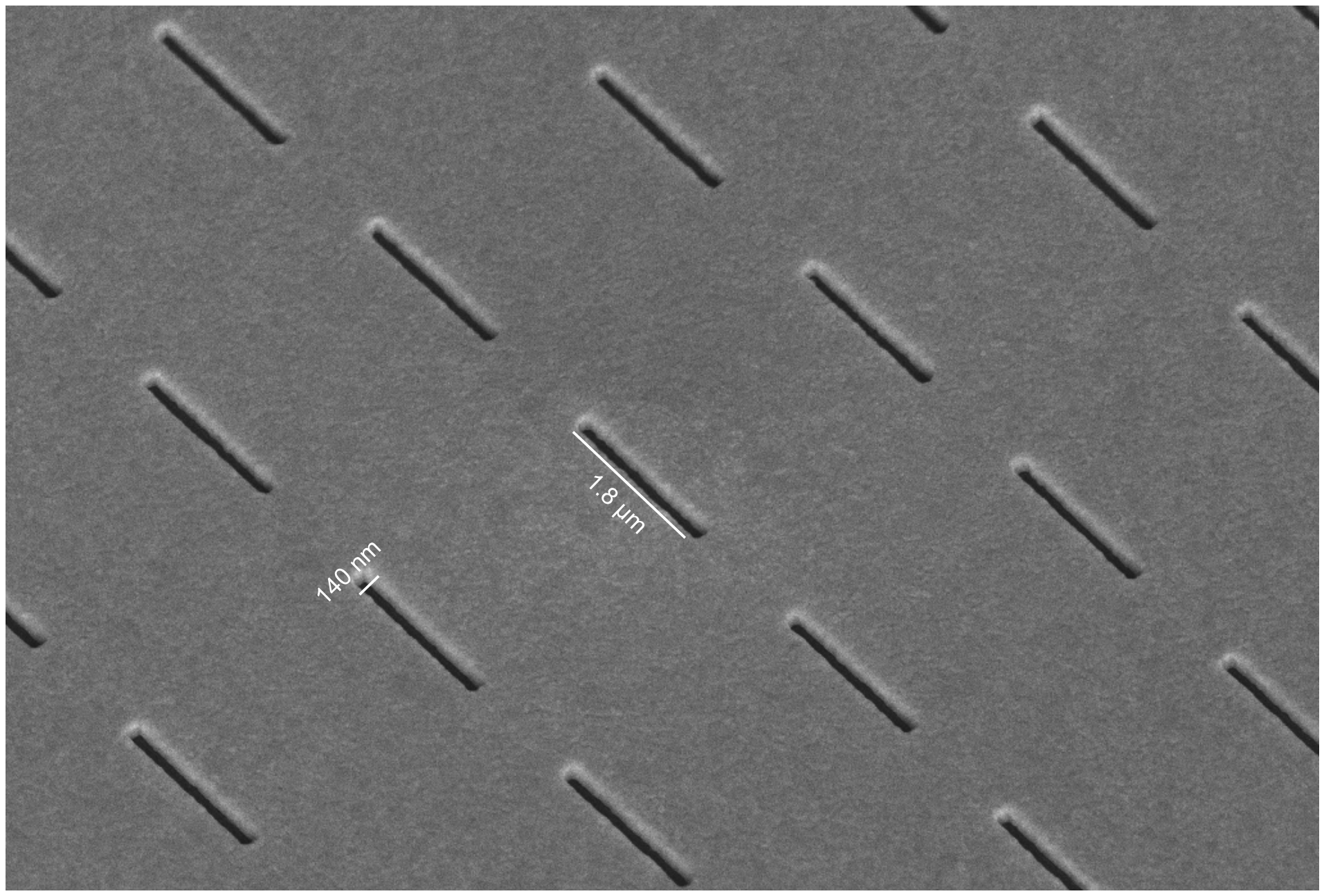

Supplementary Figure S1. Scanning electron micrograph (SEM) picture of the slit metasurface with geometric parameters to achieve 1100 cm^{-1} resonance.

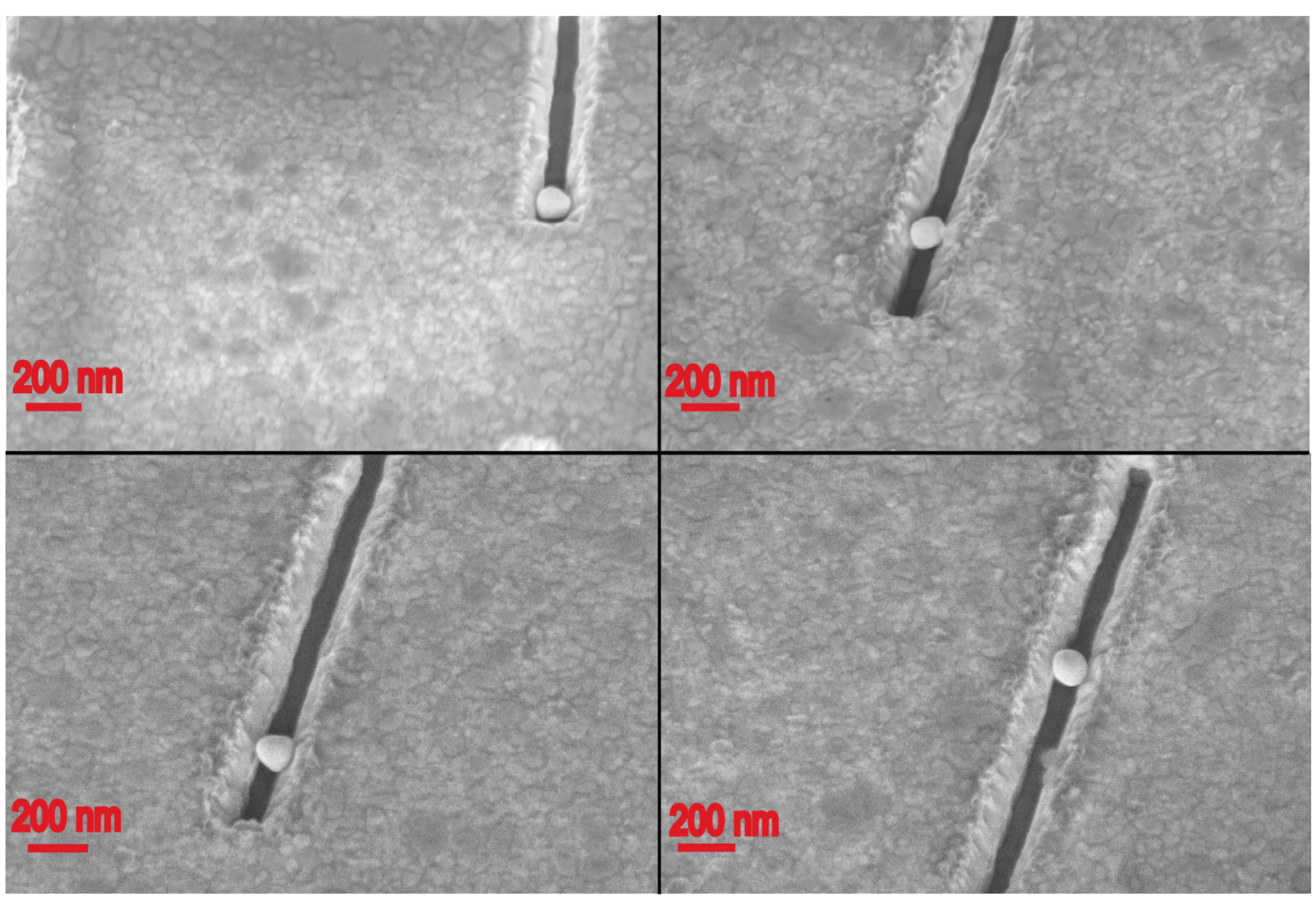

Supplementary Figure S2. Scanning electron micrograph (SEM) picture of a few representative 150 nm-diameter NPoSs.

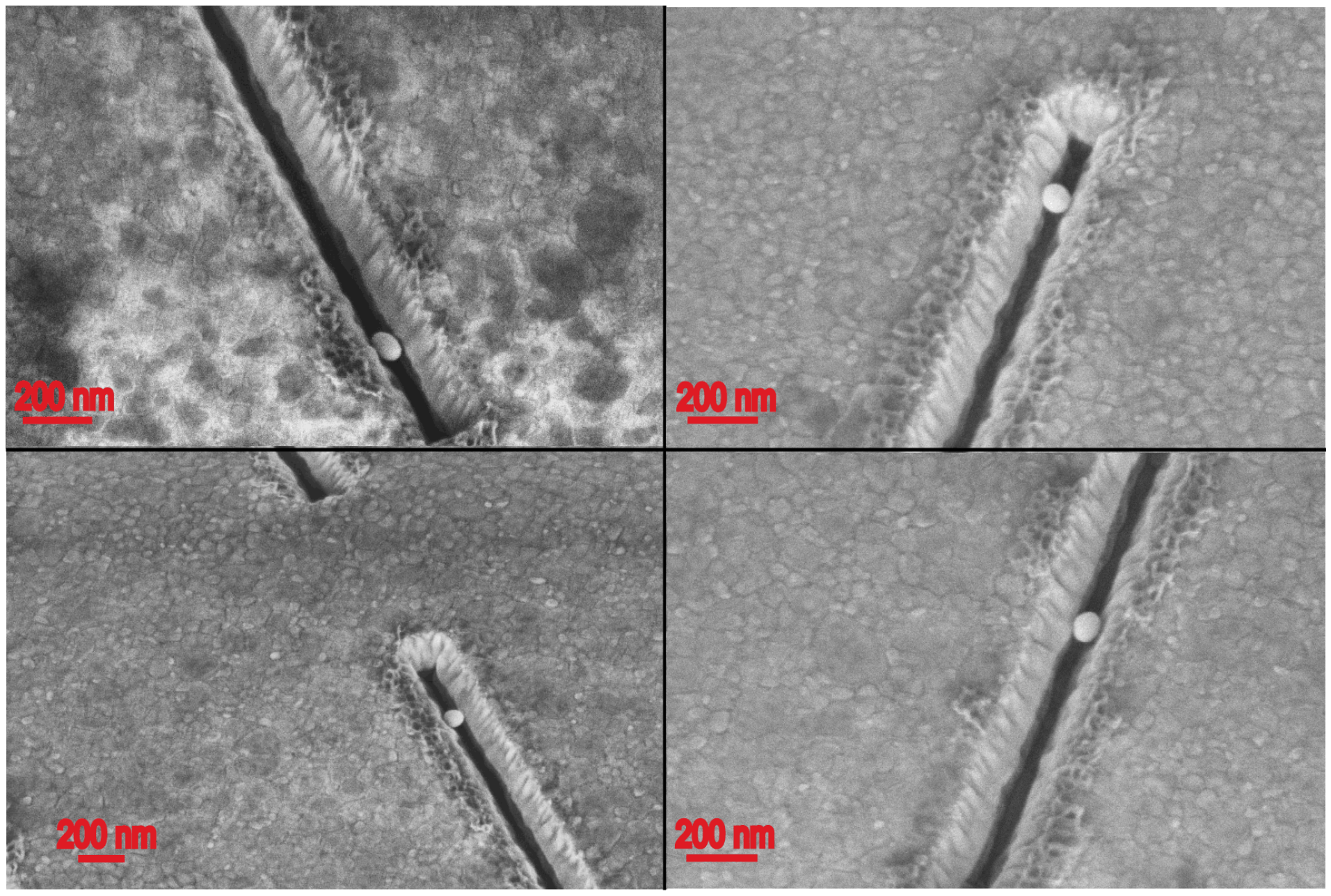

Supplementary Figure S3. Scanning electron micrograph (SEM) picture of a few representative 80 nm-diameter NPoSs.

Sec. S3. FTIR CHARACTERIZATION OF NANOSLITS ARRAY

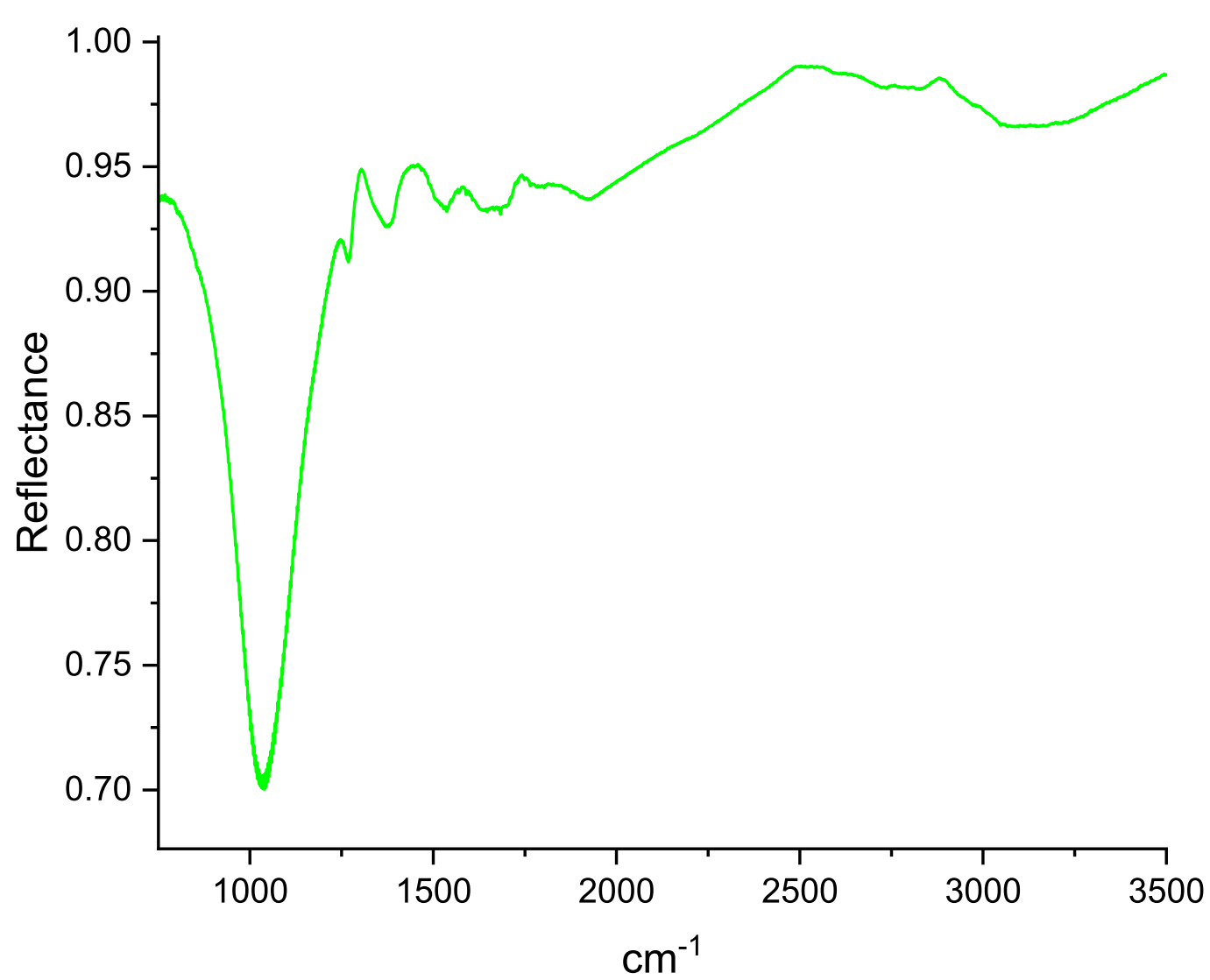

Supplementary Figure S4. Reflection spectra of the 1.8 μm slit array ,recorded using a a Bruker Vertex 80v FT spectrometer coupled to an IR microscope (HYPERION 3000) equipped with a liquid-nitrogen-cooled mercury cadmium telluride (MCT) detector. The sample was illuminated using a Cassegrain 15x objective (0.4 NA) to mildly focus IR light onto the sample surface. The reflected light was collected with the same objective. The collection area was restricted to an approximately 500μm^2 central region of the metasurface using a double-blade aperture placed in the conjugate image plane of the IR microscope. The sample chamber was continuously purged with dry air to maintain a stable, low-humidity environment.

Sec. S4. ADDITIONAL SPECTRA FROM NPOS WITH VARIOUS MOLECULAR SPACERS

The following figures present additional SERS and SFG/DFG measurements from individual NPoS on the four different types of samples discussed in the main text, Fig. 4, plus two other configurations. In each figure below, panel (a) shows a sketch of the molecular configuration in the nanogaps. The SFG (blue line) and DFG (red) spectra of a representative NPoS are plotted in panel (b) on a linear scale (right axis, normalized by the product of VIS and IR power), together with their logarithmic ratio (black line, left axis). Panel (c) presents independent results from distinct NPoS with the same molecular configuration (stacked with an offset of $10^{1.4}$), with their respective normalized SERS spectra plotted in panel (d) using the same colors.

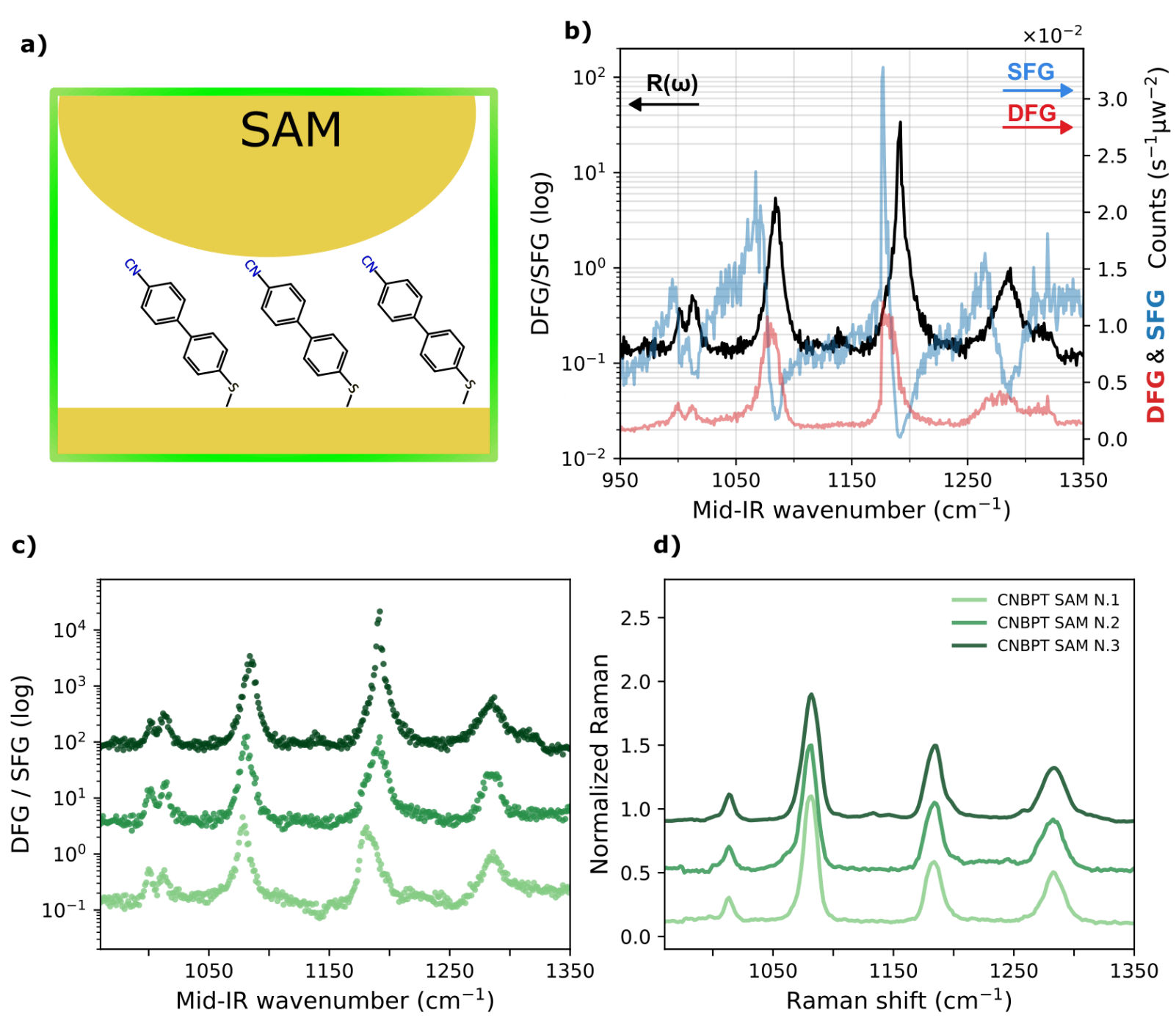

Supplementary Figure S5. Additional spectra for CN-BPT SAM on the slits.

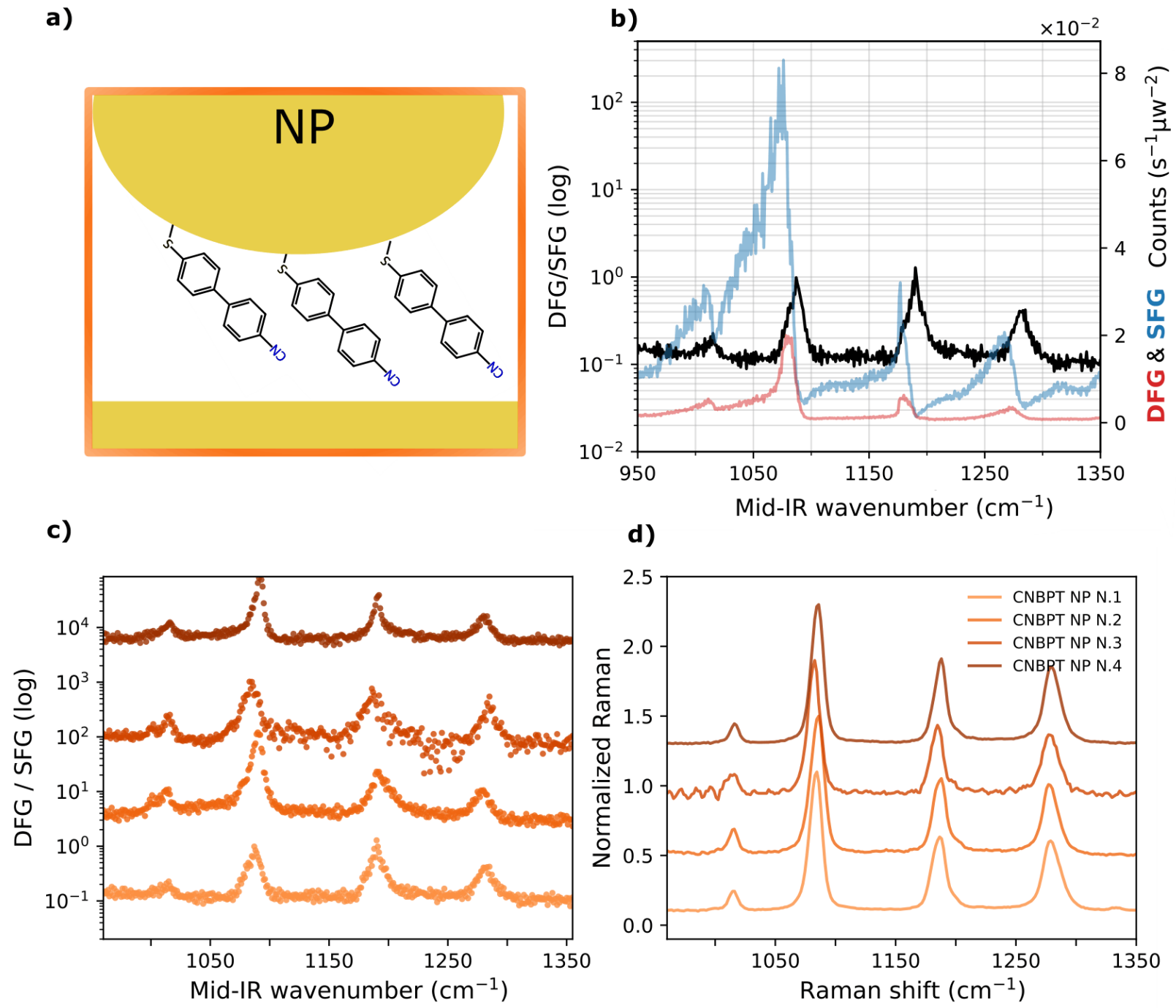

Supplementary Figure S6. Additional spectra for CN-BPT functionalized nanoparticles.

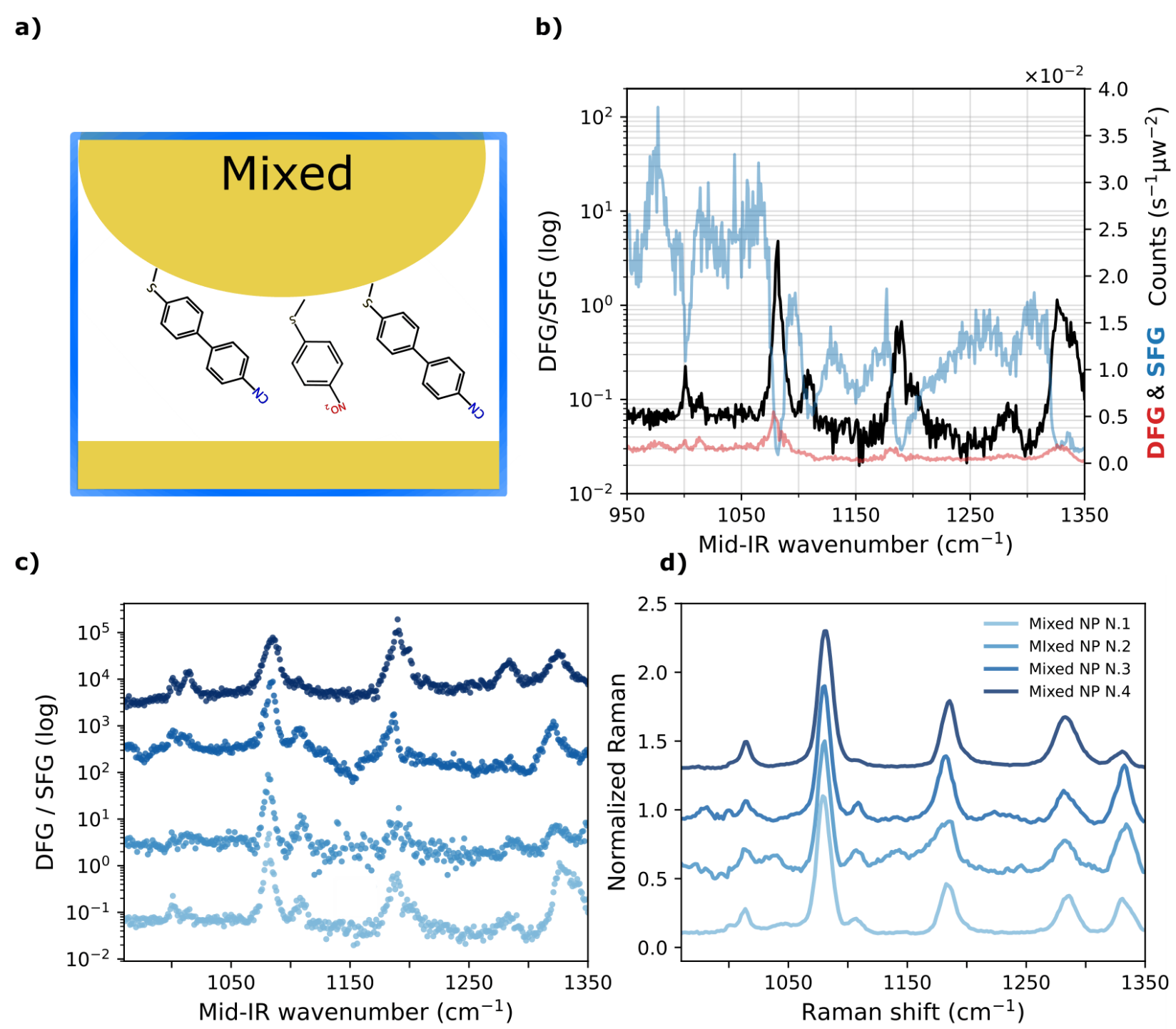

Supplementary Figure S7. Additional spectra for mixed CN-BPT / 4NPT functionalisation of the nanoparticles.

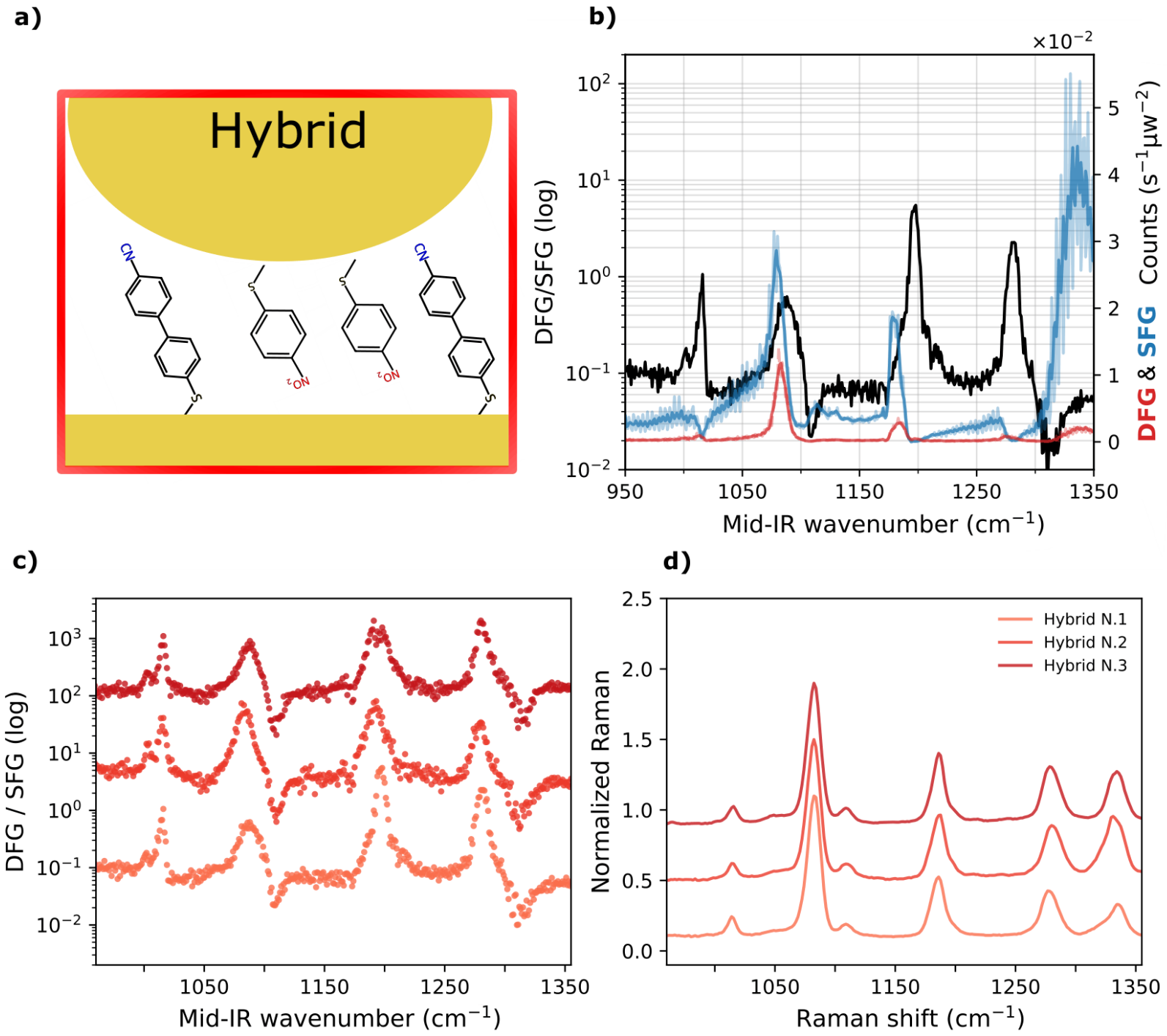

Supplementary Figure S8. Additional spectra for hybrid junctions: CN-BPT SAM on the slit, 4NTP on the nanoparticle.

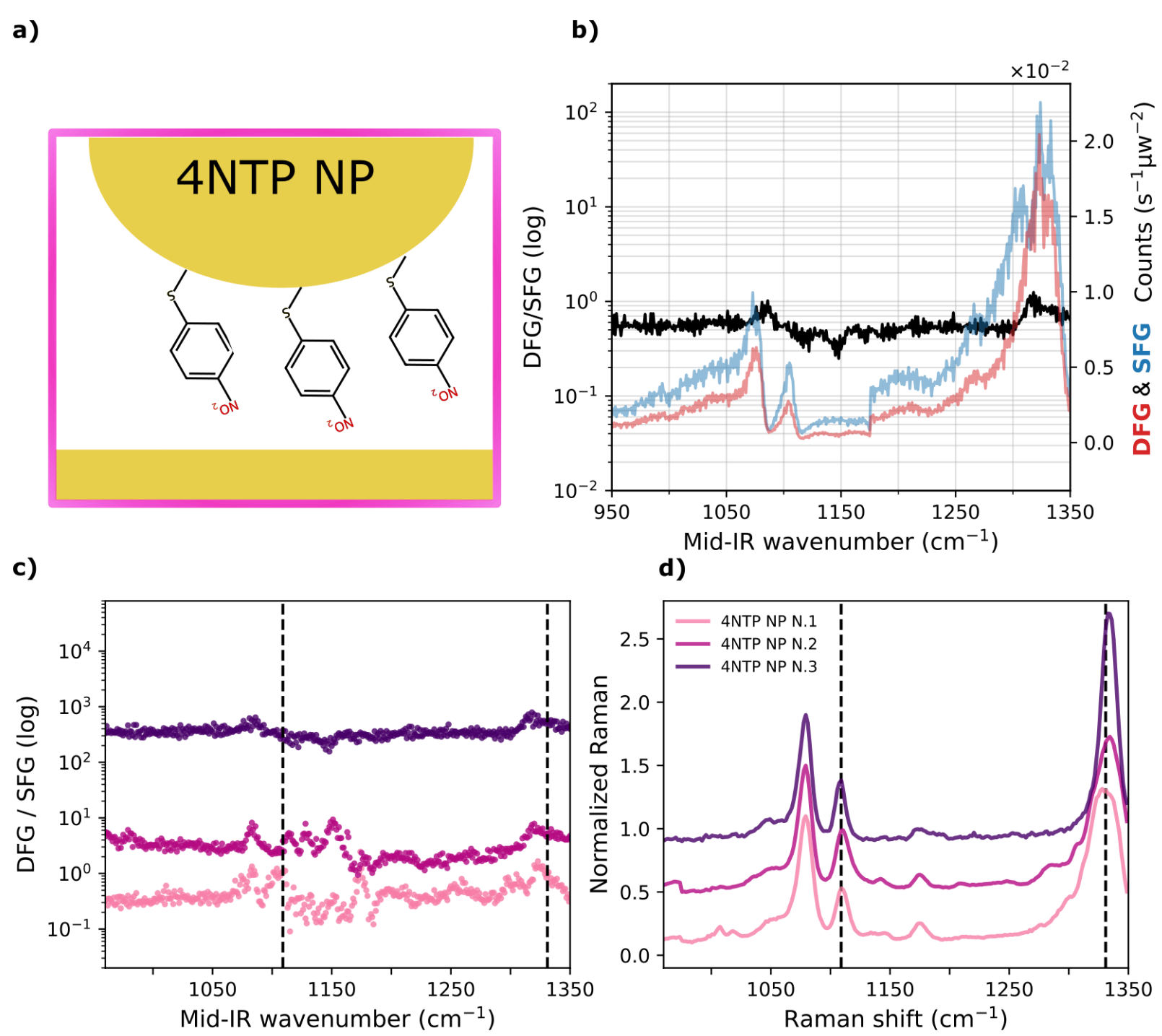

Supplementary Figure S9. Spectra for pure 4NTP samples (not shown in the main text). The very weak nonresonant $\chi^{(2)}$ in this case results in flat and featureless spectra of the DFG/SFG ratio $R(\omega)$.

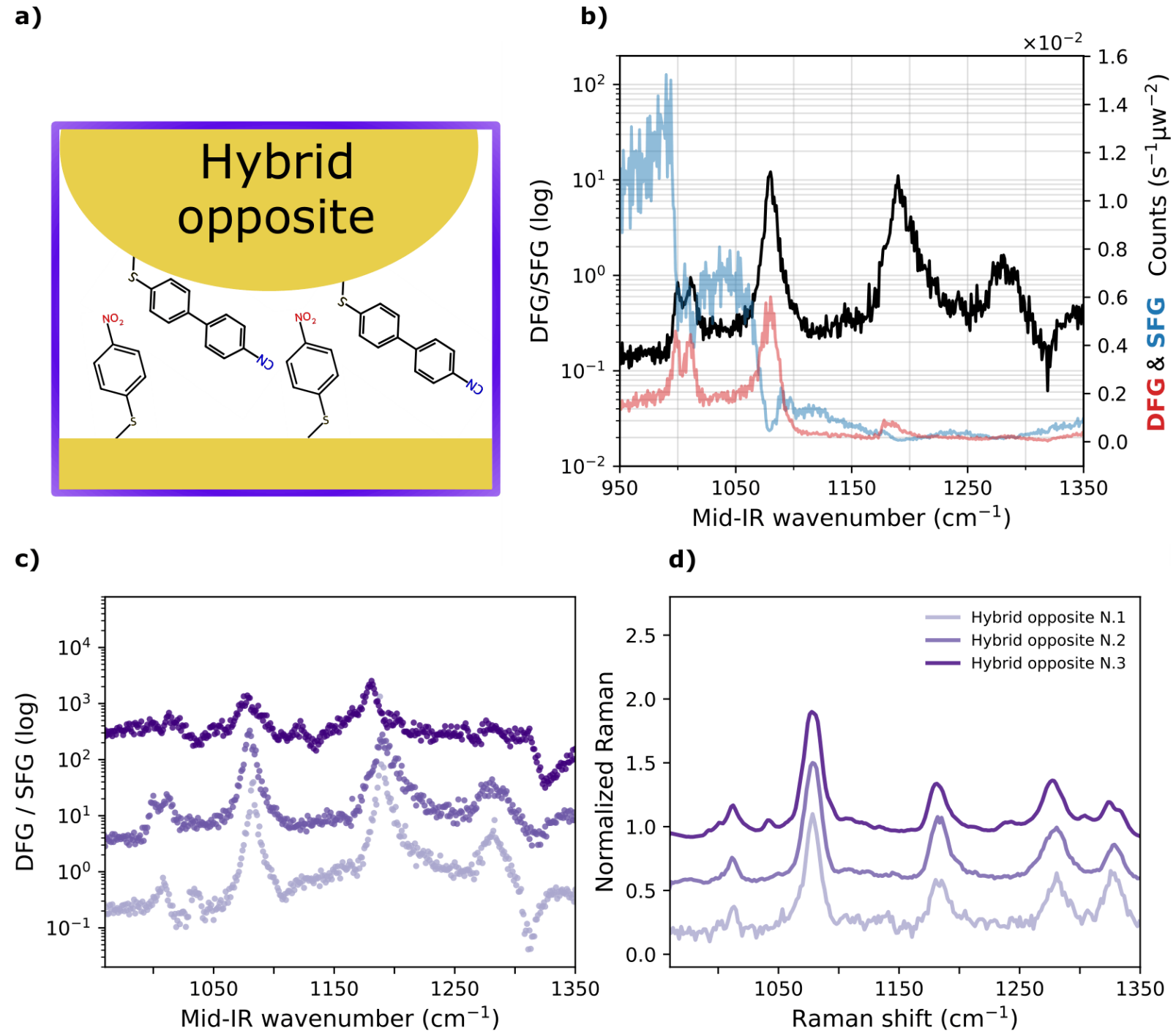

Supplementary Figure S10. Spectra for reversed hybrid junctions (not shown in main text): 4NTP SAM on the slit, CN-BPT on the nanoparticle.

Sec. S5. SERS QUANTIFICATION OF THE MOLAR RATIO OF MIXED SAM

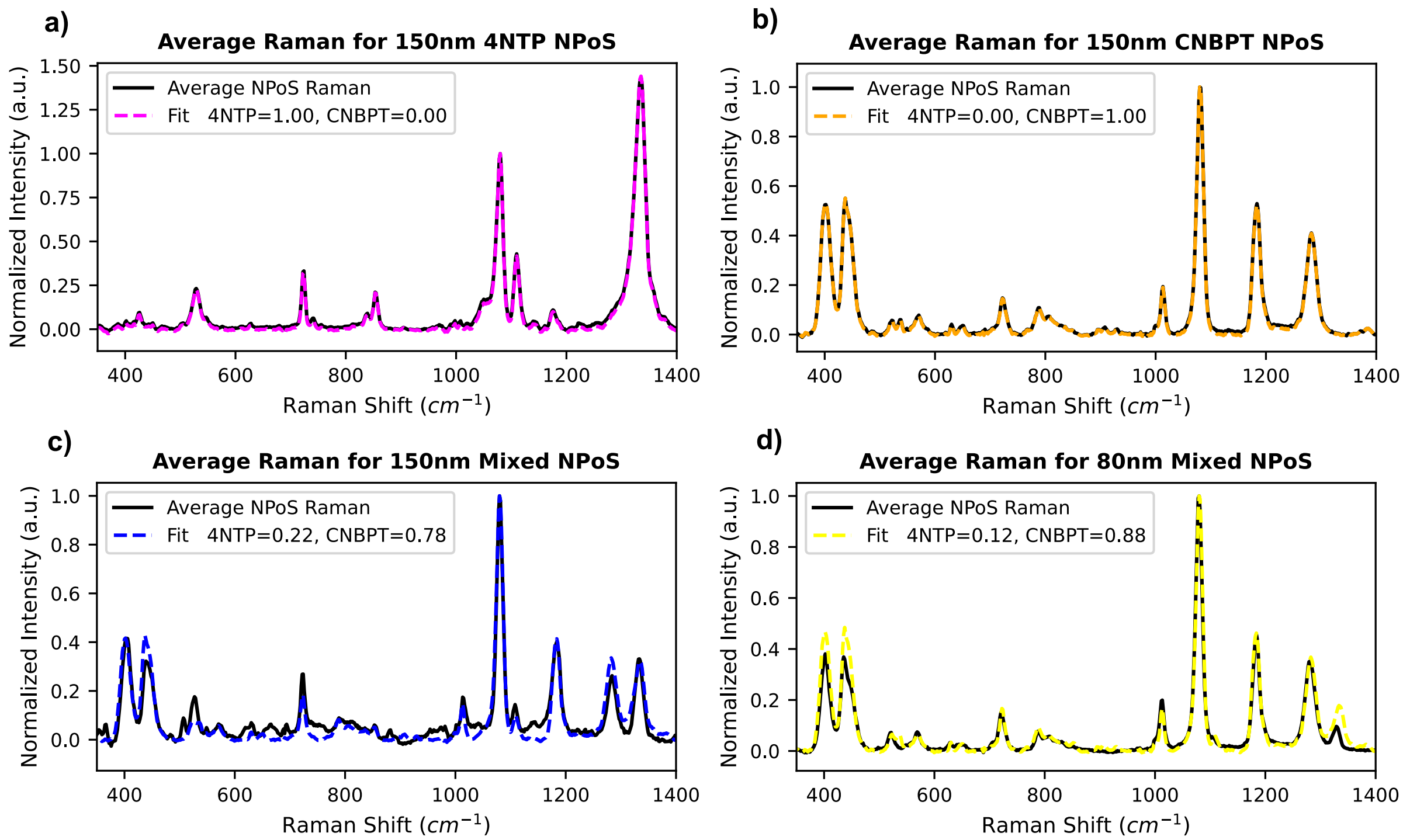

Supplementary Figure S11. (a) Average SERS spectrum of 4NTP in NPoS, and (b) of CNBPT in NPoS, used as the basis set for the linear combination. (c,d) Average SERS spectrum of (c) 150 nm-diameter and (d) 80 nm-diameter mixed-SAM NPoS; the molar ratio shown in the legend is extracted by fitting it with a linear combination of (a) and (b).

To quantify the relative population of 4NTP and CNBPT molecules in the mixed-SAM NPoS, we adopted the approach used in [1, 2]: we first collected the SERS signal from a dozen pure 4NTP and pure CNBPT cavities, and after normalization for the common peak at 1080 cm^{-1}, we calculated the average SERS spectra for each of the two groups of NPoS (Figs. S11a,b). The average spectra are used as the two references for a linear combination fit where the coefficients are identified with the molar ratio of each molecule in the SAM. The fitting curves for the samples of Fig. S7 and Fig. S13 are shown with dashed lines in Fig. S11c and d, respectively.

Sec. S6. NPOS STABILITY UNDER LASER EXPOSURE

An advantage of using continuous-wave excitation for both the MIR and VIS is that it significantly increases molecular stability compared to pico- or femtosecond pulsed experiments where the molecules experience high transient fields. Typically, a safe operating regime for us is at 5 μW VIS and 10 mW MIR powers; under these conditions, most of the cavities are stable, and experiments are reproducible, as shown for 80 nm NPoS in Fig. S12. Thermal stability is a limitation at high MIR powers: the visible power can be increased to more than 20 μW without deteriorating the spectrum; but when the MIR laser is tuned resonantly to a molecular vibration, absorption is increased, and we

sometimes observe bleaching of the signal attributed to excess heat for powers above 10 mW. Still, we note that the gold film is a good thermal conductor, making our design particularly resilient to thermal hotspots.

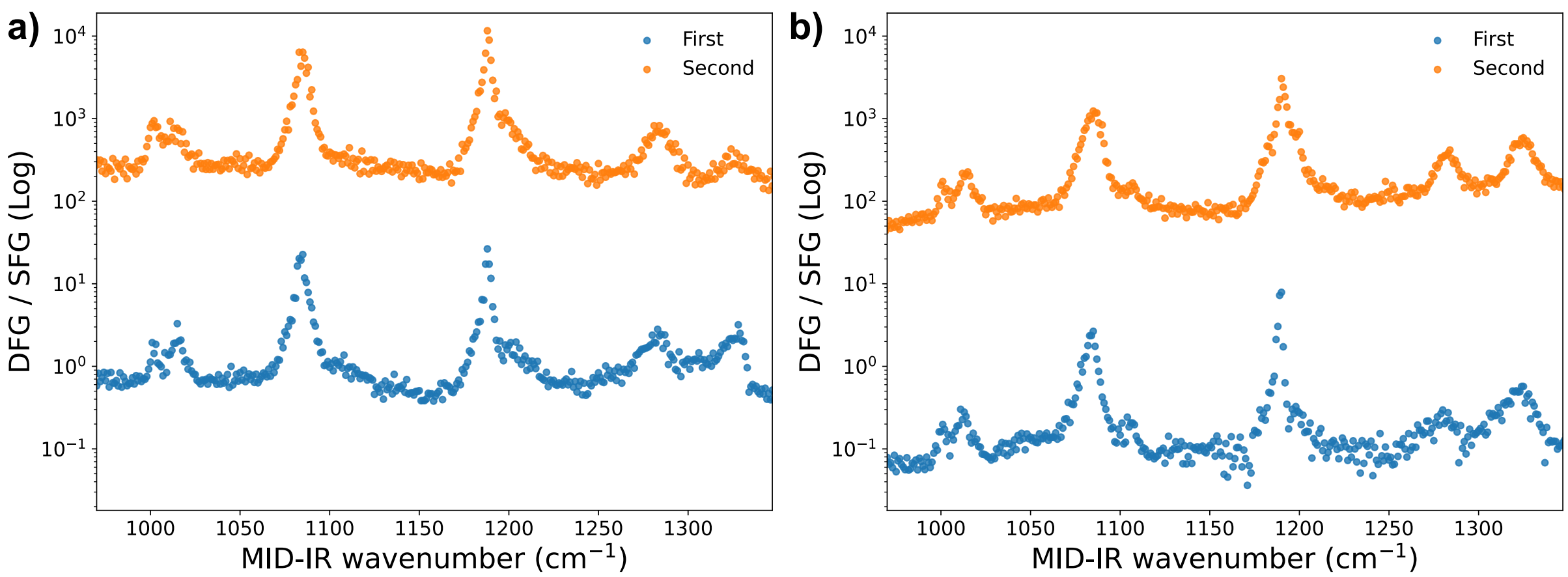

Supplementary Figure S12. (a) Ratiometric spectra for a single 80 nm mixed-SAM NPoS, before (blue) and after (orange) one hour of continuous VIS and MIR exposure (b) Ratiometric spectra of another single 80 nm Mixed NPoS, before (blue) and after (orange) one hour of continuous VIS and MIR exposure. The curves are arbitrarily offset for clarity.

Sec. S7. ESTIMATION OF THE DETECTION LIMIT

In order to estimate the limit of detection of the technique, i.e., how many molecules can be reliably probed, we fabricated a second mixed SAM NPoS sample by decreasing the size of the nanoparticles dropcasted from 150 nm to 80 nm. According to Ref. [3], the average number of biphenyl-4,4'-dithiol(BPDT) molecules under a 60 nm Au NPoM was estimated to be 176 for an active area of 35 nm^2, thanks to conductance measurements performed on the nanogap. Also, in Ref. [4], the authors estimated a similar mode area for a 60 nm particle. Assuming that the active area scales as the square of the nanoparticle radius, and for a similar packing density of biphenyl-dithiol and CN-BPT, we calculated that $\approx$ 315 thiol molecules are trapped under an 80 nm NPoM. Considering an NPoS system, we expect to have two times more molecules since the nanoparticle touches the slit on two sides. To further challenge our technique, we decreased the surface fraction of 4NTP in the mixed SAM down to $\approx$ 10% so that the 4NTP population within the gap is of the order of **60 molecules**. Despite the reduced number of molecules in the gap, we could still retrieve the molecular orientation through the logarithmic ratio: a representative spectrum of a 80 nm mixed-SAM NPoS is displayed in Fig. S13; it exhibits the two peaks in $R(\omega)$ at 1109 and 1331 cm^{-1}, as expected for 4NTP when it is anchored on the same surface as CN-BPT.

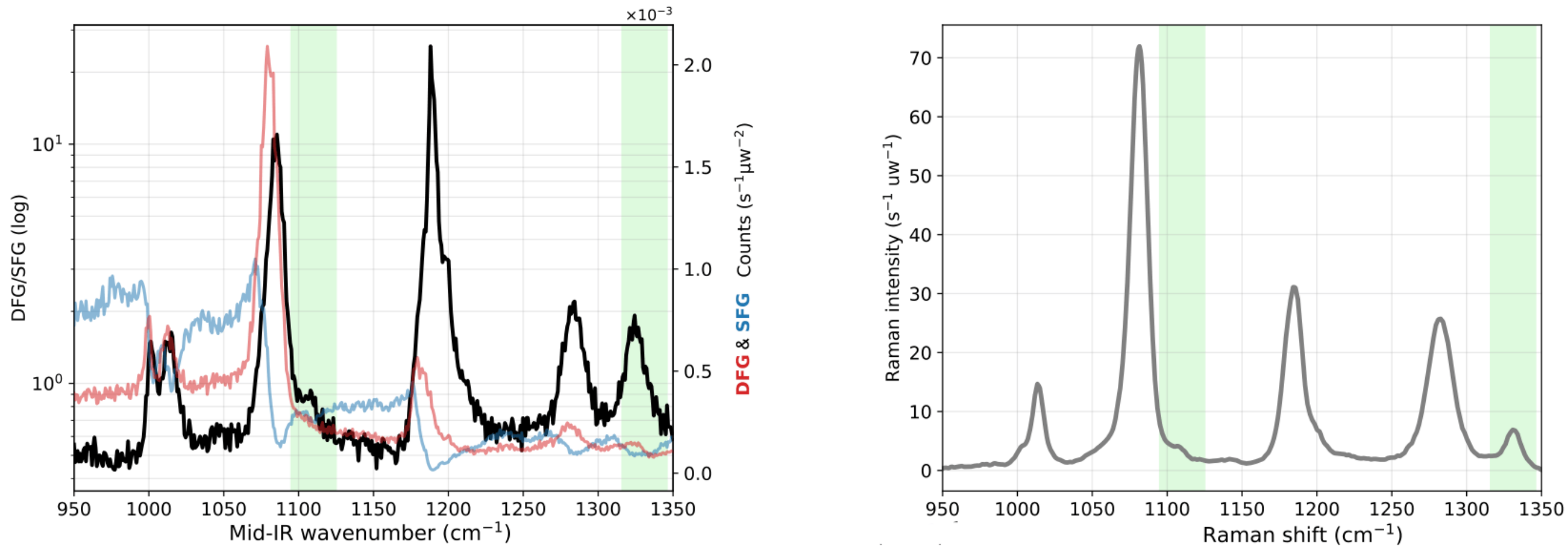

Supplementary Figure S13. **(a)** SFG (blue line) and DFG (red) spectra of a single representative 80 nm mixed-SAM NPoS are reported on a linear scale on the right axis, together with their logarithmic ratio (black line, left axis). The spectra are normalized by the product of MIR and VIS power; the acquisition time is 2 s per MIR wavenumber. **(b)** Normalized SERS spectra for the same NPoS. The infered number of probed 4NTP molecules is ≈ 60.

Sec. S8. MORE COMPLETE MODEL OF NPOS NON-LINEAR RESPONSE

As explained in the main text and in Sec. S1, two identical molecules oriented antiparallel to each other and driven by common VIS and MIR local fields exhibit second-order SFG and DFG polarisations that are in opposition of phase. A similar cancellation may occur between identical molecules located in the two opposite nanogaps of the NPoS structure, if these two gaps are driven in phase by the local fields. This cancellation could be total for a perfectly centrosymmetric NPoS geometry, as expected by the general selection rule. However, several geometrical factors break the inversion symmetry of the NPoS: the presence of the substrate, the strongly slanted sidewalls, the deviation of the nanoparticle shape from a sphere, the asymmetry in the particle-wall contacts, etc. (see Figs. S2 and S3). As illustrated in Fig. S14, the slanted sidewalls alone prevent the cancellation of the vertical component of the nonlinear dipole in this situation. Thus, the extent of symmetry breaking in the NPoS only changes the total signal strength (indeed, SFG/DFG are very weak for vertical sidewalls, as reported in [5]). In contrast, the sign of the vibrational peaks relative to the background and to each other is insensitive to these geometrical factors, as long as the molecular configuration remains homogeneous across both nanogaps.

These considerations can be formalized by introducing a spatially varying nonlinear susceptibility tensor $\chi^{(2)}(\vec{r})$ in the mode overlap integral used in [6] and many other similar theoretical studies, with the nonlinear signal strength being proportional to the modulus square of the contracted volume integral (implicit summation on repeated indices)

$$\int dV\, \chi^{(2)}_{ijk}(\boldsymbol{r}) e^{\mathrm{MIR}}_i(\boldsymbol{r}) e^{\mathrm{VIS}}_j(\boldsymbol{r}) e^{\mathrm{SFG}}_k(\boldsymbol{r}) \tag{1}$$

in which the near-field distributions $\boldsymbol{e}(\boldsymbol{r})$ at all involved wavelengths (MIR and VIS lasers, SFG signal) appear. The strength of the SFG signal therefore depends on the symmetry of the combined plasmonic modes and $\chi^{(2)}(\vec{r})$. A

detailed discussion of the quasi-normal modes (QNMs) supported by NPoS structures, including their near fields and far-field radiation patterns, is available in [6].

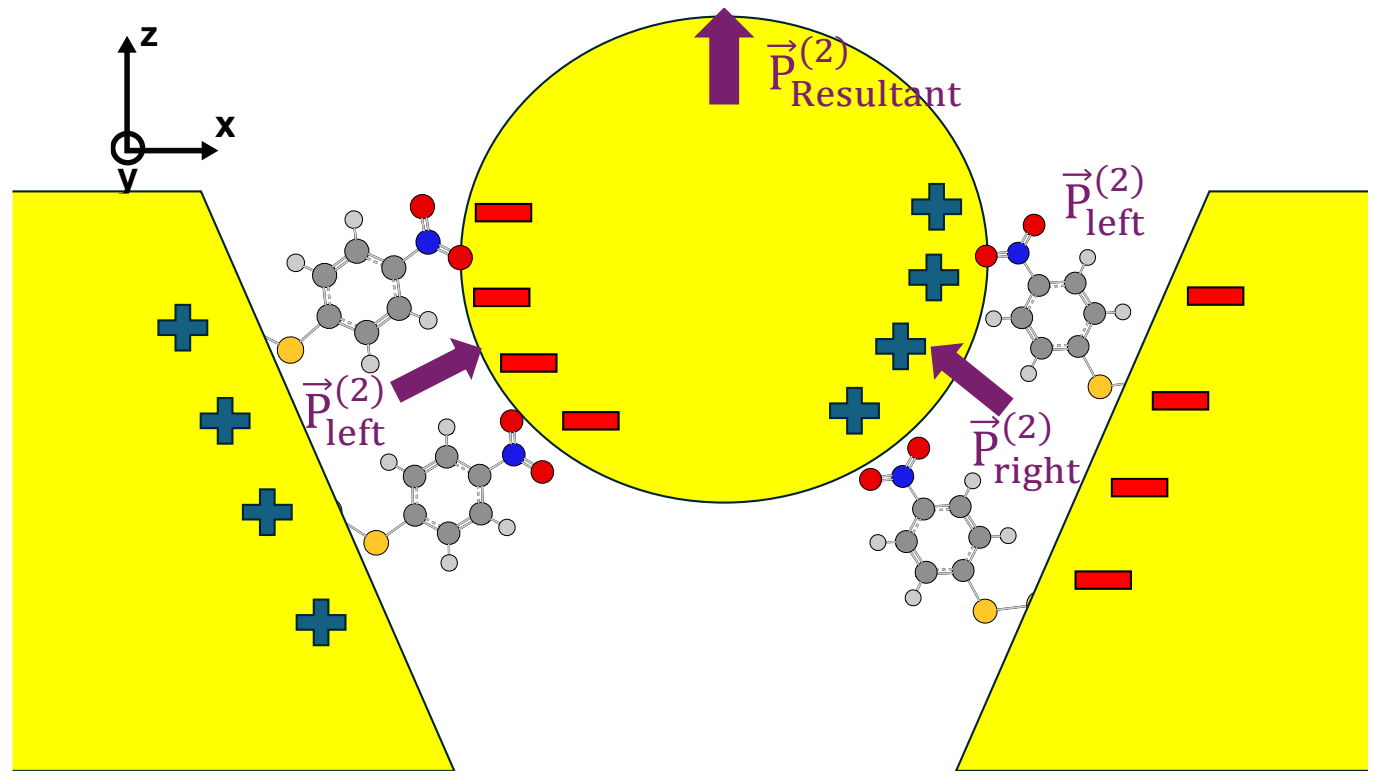

Supplementary Figure S14. Sketch of the nonlinear dipoles in the NPoS system. We assume that both the MIR and VIS incident fields couple to plasmonic modes having odd mirror symmetry with respect to a (y, z) plane containing the nanoparticle diameter, as illustrated by the instantaneous charge distribution. If a molecular species is functionalized on the nanoparticle, or on the substrate, its $\chi^{(2)}$ response will be even under this symmetry, and so will be the induced second-order polarization $P^{(2)}$ (purple arrows). The interference between the two nanogaps may result in the cancellation of the (x, y) in-plane components, but the z components add up.

Sec. S9. FITTING PARAMETERS

The fitting procedure is as follows: the non-resonant DFG/SFG ratio is extracted by averaging the signal over small frequency ranges far from any vibrational peak. For Fig. 4 in the main text, the phase of the 1080 cm^{-1} peak is kept fixed at 0° while all the others are forced to be either $180° \pm 5°$ or $0° \pm 5°$. The obtained parameters are listed in Table S1. Constraining the phase to be exactly π or 0 doesn't quantitatively change the quality of the fit except for the peak at 1331 cm^{-1} (see Fig. S15 and Table S2). The non-resonant background amplitude A_{nr} is arbitrarily fixed to unity. The width of vibrational peaks is allowed to vary between 3 and 8 cm^{-1} since the linewidth of SFG and DFG peaks is not always the same as those of the corresponding Raman peaks [1]. The peak positions are fixed by the Raman peaks. For a highly IR-active dipole mode such as the NO_2 stretching, the IR coupling causes a collective mode splitting with a new peak at 1320 cm^{-1} observed by Bell et al. [1]. Slightly relaxing the constraint on ϕ_{1331} or including this new mode explicitly helps improve the fit, but brings no new insights about molecular orientation.

Supplementary Table S1. Fit Parameters for the four spectra of Fig. 4 (Phases in degrees).

**Parameter**	**CNBPT NP**	**CNBPT SAM**	**Mixed NP**	**Hybrid**
A_{1001}	0.7716	1.5974	2.6700	3.0000
A_{1014}	0.6737	1.2805	1.4400	1.6600
A_{1080}	5.3689	5.1027	7.0700	6.4500
A_{1109}	–	–	1.7300	1.9400
A_{1185}	5.6318	7.0196	10.5000	12.6000
A_{1281}	2.6671	4.1133	0.5890	2.7400
A_{1331}	–	–	12.0000	21.8000
ϕ_{1001} (°)	0.18°	−2.70°	−5.00°	−1.51°
ϕ_{1014} (°)	−3.60°	−3.06°	0.00°	−1.10°
ϕ_{1080} (°)(fixed)	0.00°	0.00°	0.00°	0.00°
ϕ_{1109} (°)	–	–	0.00°	175.00°
ϕ_{1185} (°)	5.04°	5.04°	5.00°	1.33°
ϕ_{1281} (°)	−3.24°	0.54°	−5.00°	−4.96°
ϕ_{1331} (°)	–	–	5.00°	185.00°
γ_{1001} (cm^{-1})	6.8217	3.9300	8.0000	7.9500
γ_{1014} (cm^{-1})	4.0926	3.2919	8.0000	3.0500
γ_{1080} (cm^{-1})	7.9752	6.2952	8.0000	8.0000
γ_{1109} (cm^{-1})	–	–	6.0400	5.3400
γ_{1185} (cm^{-1})	7.9977	7.9999	6.0800	7.4300
γ_{1281} (cm^{-1})	7.2399	7.8191	4.8200	6.8900
γ_{1331} (cm^{-1})	–	–	8.5000	6.3000
DFG/SFG ratio	0.1107	0.1649	0.0405	0.1662
A_{nr} (fixed)	1.0000	1.0000	1.0000	1.0000
ϕ_{nr} (°)	49.32°	59.22°	80.20°	47.80°

Supplementary Table S2. Fit Parameters for the four spectra of Fig. S15 (Phases in degrees).

**Parameter**	**CNBPT NP**	**CNBPT SAM**	**Mixed NP**	**Hybrid**
A_{1001}	1.0600	1.5300	1.8600	2.1900
A_{1014}	0.7320	1.5400	0.7230	2.0700
A_{1080}	6.1500	7.5100	7.1900	5.9200
A_{1109}	–	–	0.9210	2.0000
A_{1185}	8.9100	12.5000	7.1800	7.3500
A_{1281}	2.8300	4.0700	0.1000	2.3900
A_{1331}	–	–	5.5400	3.6700
ϕ_{1001} ($^\circ$)	$0.^\circ$	0°	0°	0°
ϕ_{1014} ($^\circ$)	0°	0°	0°	0°
ϕ_{1080} ($^\circ$)(fixed)	0°	0°	0°	0°
ϕ_{1109} ($^\circ$)	–	–	0°	180°
ϕ_{1185} ($^\circ$)	0°	0°	0°	0°
ϕ_{1281} ($^\circ$)	0°	0°	0°	0°
ϕ_{1331} ($^\circ$)	–	–	0°	180°
γ_{1001} (cm^{-1})	8.0000	4.0700	8.0000	5.1000
γ_{1014} (cm^{-1})	3.8100	4.0300	8.0000	3.4000
γ_{1080} (cm^{-1})	8.0000	5.1600	5.6000	7.1700
γ_{1109} (cm^{-1})	–	–	3.7100	5.2300
γ_{1185} (cm^{-1})	3.5300	8.0000	3.8100	7.9200
γ_{1281} (cm^{-1})	7.6500	8.0000	2.0800	4.9200
γ_{1331} (cm^{-1})	–	–	8.0000	7.7500
DFG/SFG ratio	0.1093	0.1380	0.0502	0.1456
$A_{\rm nr}$ (fixed)	1.0000	1.0000	1.0000	1.0000
$\phi_{\rm nr}$ ($^\circ$)	45.51°	79.59°	81.00°	44.88°

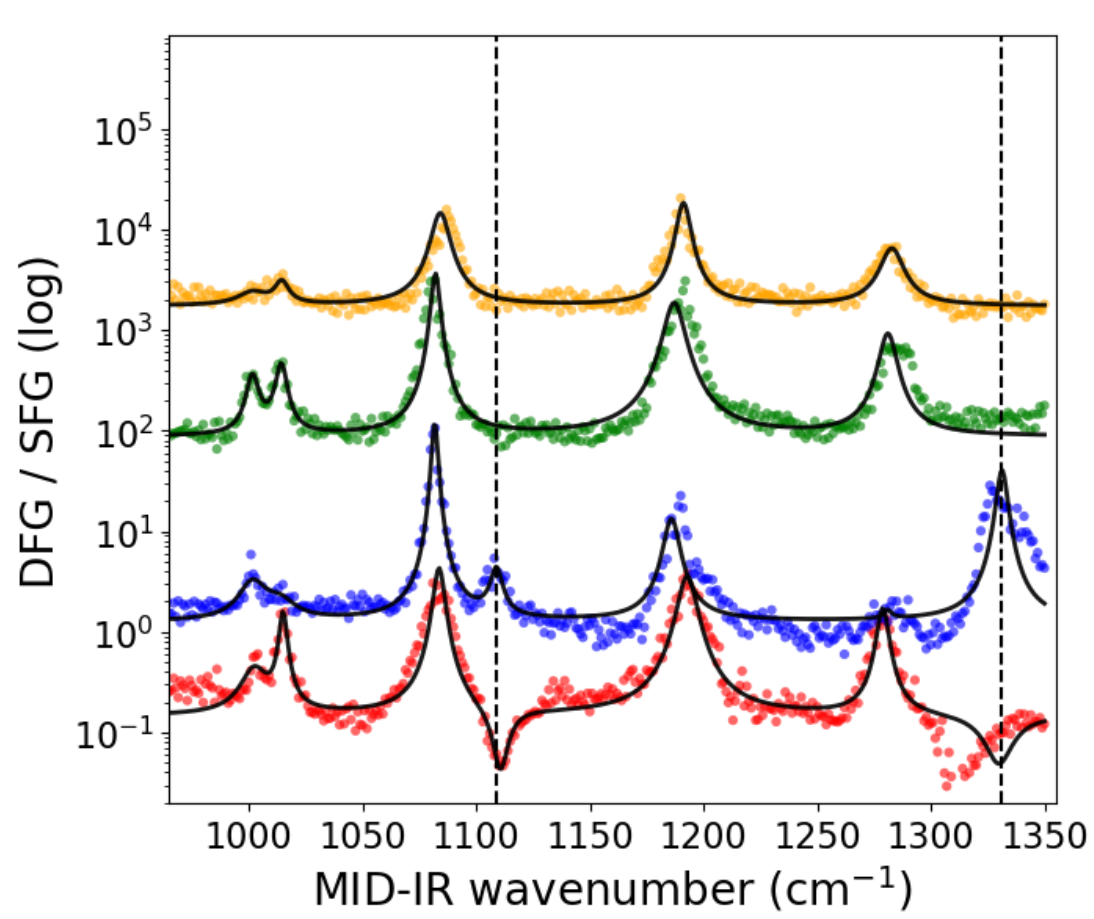

Supplementary Figure S15. Same ratiometric plot as Fig. 4; the only difference is that in the fits (black solid curves) ϕ_i is forced to be either exactly π or 0.

Sec. S10. VIBRATIONAL PATTERNS OF RELEVANT MODES

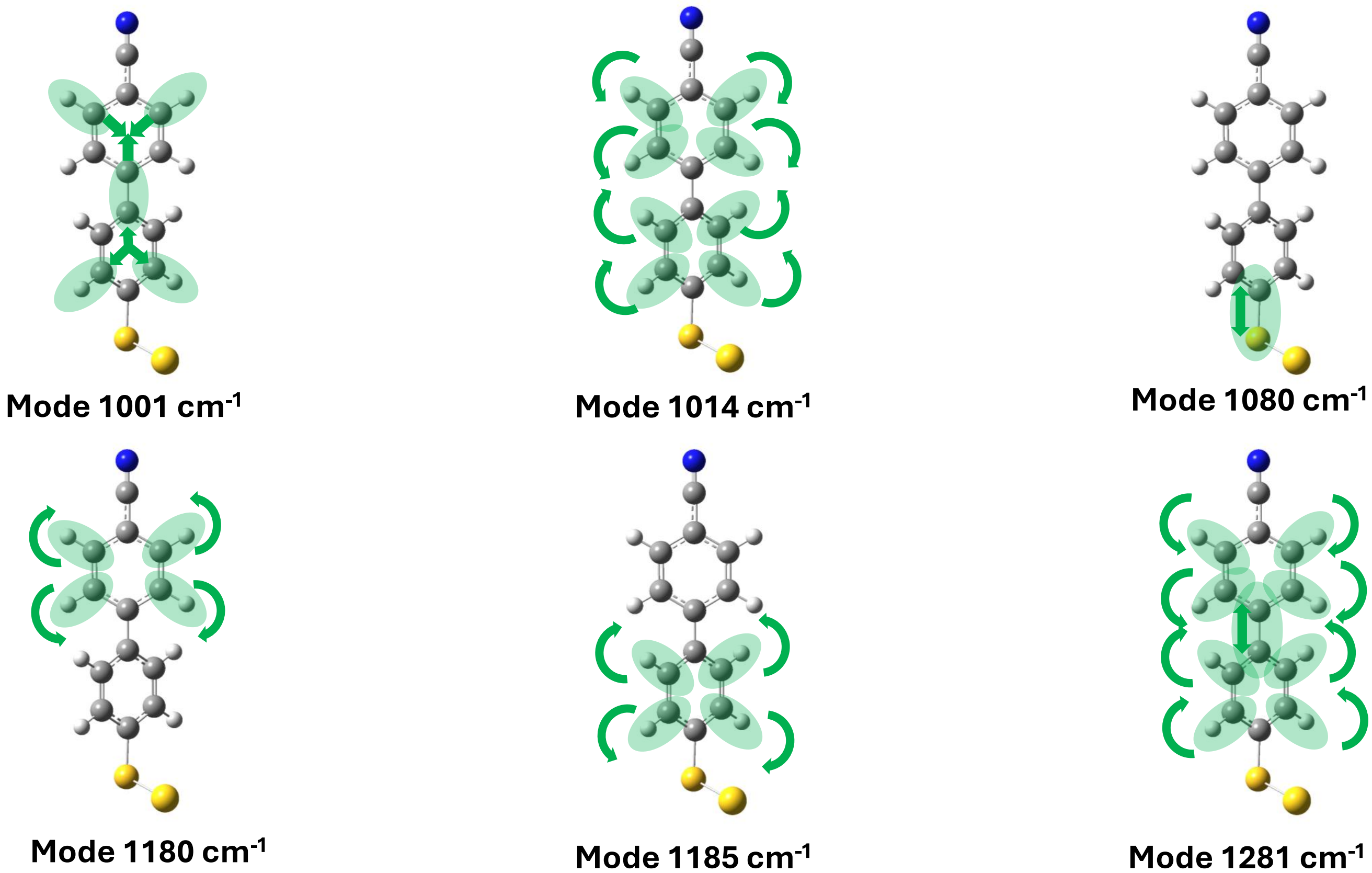

Supplementary Figure S16. Atomic displacements corresponding to the relevant CNBPT vibrational modes in this study.

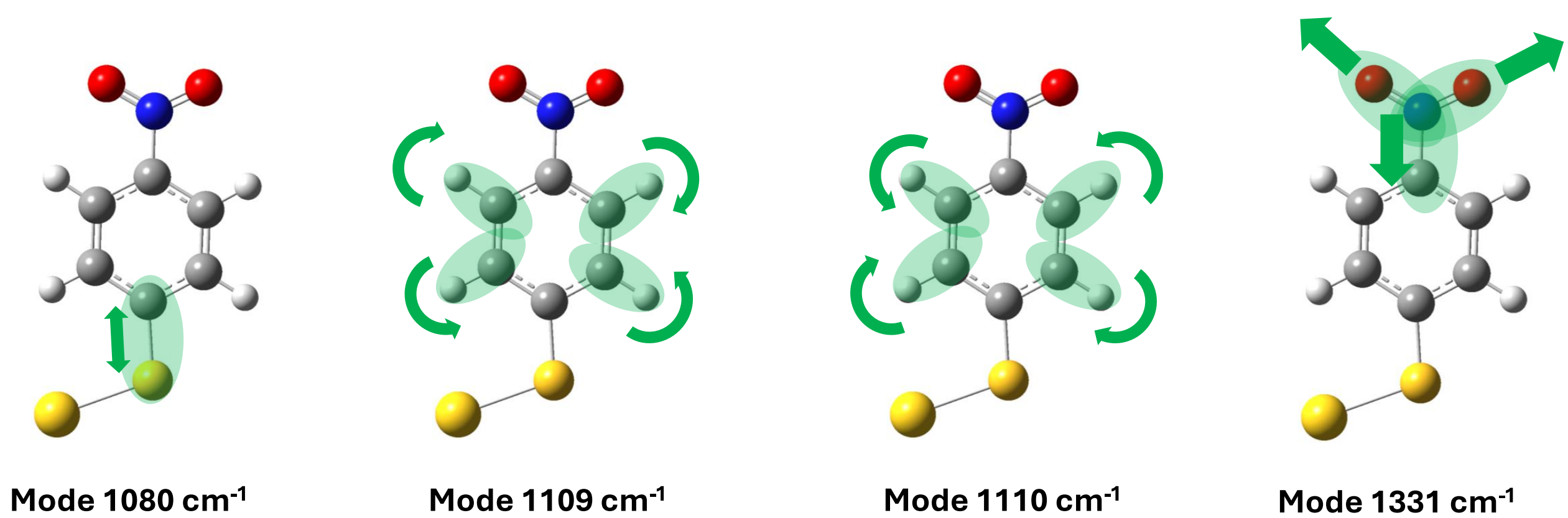

Supplementary Figure S17. Atomic displacements corresponding to the relevant 4NTP vibrational modes in this study.

[1] F. Bell, L. Jakob, C. Todd, I. Lohia, Y. Roh, R. Arul, and J. J. Baumberg, "Coherent Dynamics of Molecular Vibrations in Single Plasmonic Nanogaps," *Physical Review Letters*, vol. 135, p. 076901, Aug. 2025.

[2] N. S. Mueller, R. Arul, L. A. Jakob, M. O. Blunt, T. Földes, E. Rosta, and J. J. Baumberg, "Collective Mid-Infrared Vibrations in Surface-Enhanced Raman Scattering," *Nano Letters*, vol. 22, pp. 7254–7260, Sept. 2022.

[3] F. Benz, C. Tserkezis, L. O. Herrmann, B. de Nijs, A. Sanders, D. O. Sigle, L. Pukenas, S. D. Evans, J. Aizpurua, and J. J. Baumberg, "Nanooptics of Molecular-Shunted Plasmonic Nanojunctions," *Nano Letters*, vol. 15, pp. 669–674, Jan. 2015.

[4] R. Chikkaraddy, B. de Nijs, F. Benz, S. J. Barrow, O. A. Scherman, E. Rosta, A. Demetriadou, P. Fox, O. Hess, and J. J. Baumberg, "Single-molecule strong coupling at room temperature in plasmonic nanocavities," *Nature*, vol. 535, pp. 127–130, July 2016.

[5] W. Chen, P. Roelli, H. Hu, S. Verlekar, S. P. Amirtharaj, A. I. Barreda, T. J. Kippenberg, M. Kovylina, E. Verhagen, A. Martínez, and C. Galland, "Continuous-wave frequency upconversion with a molecular optomechanical nanocavity," *Science*, vol. 374, pp. 1264–1267, Dec. 2021.

[6] H. Hu, Z. Hu, C. Galland, and W. Chen, "Plasmonic Nanoparticle-on-Nanoslit Antenna as Independently Tunable Dual-Resonant Systems for Efficient Frequency Upconversion," *Advanced Optical Materials*, vol. 13, no. 29, p. e01674, 2025.
